# Effect of bleaching gels with different thickeners under normal and hyposalivation conditions: *in situ* study*

**DOI:** 10.1590/1678-7757-2022-0285

**Published:** 2022-12-02

**Authors:** Laura Nobre Ferraz, Isabele Vieira, Gláucia Maria Bovi Ambrosano, Márcio Ajudarte Lopes, Débora Alves Nunes Leite Lima

**Affiliations:** 1 Universidade Estadual de Campinas Faculdade de Odontologia de Piracicaba Departamento de Odontologia Restauradora Piracicaba SP Brasil Universidade Estadual de Campinas, Faculdade de Odontologia de Piracicaba, Departamento de Odontologia Restauradora, Piracicaba, SP, Brasil.; 2 Universidade Estadual de Campinas Faculdade de Odontologia de Piracicaba Departamento de Odontologia Social Piracicaba SP Brasil Universidade Estadual de Campinas, Faculdade de Odontologia de Piracicaba, Departamento de Odontologia Social, Piracicaba, SP, Brasil.; 3 Universidade Estadual de Campinas Faculdade de Odontologia de Piracicaba Departamento de Diagnóstico Oral Piracicaba SP Brasil Universidade Estadual de Campinas, Faculdade de Odontologia de Piracicaba, Departamento de Diagnóstico Oral, Piracicaba, SP, Brasil.

**Keywords:** Tooth bleaching, Thickeners, Salivary flow rate, Dental enamel, Hardness

## Abstract

Bleaching gel thickeners induce important changes in tooth enamel and these changes are reversed by saliva.

**Objective:**

This *in situ* study aimed to evaluate the effect of bleaching gels with different thickeners on tooth enamel under normal and hyposalivation conditions.

**Methodology:**

Of 28 participants, 14 had normal salivary flow and 14 had low salivary flow. For each salivary flow, four types of treatment were performed with different thickeners: no bleaching (negative control), bleaching with a commercial 10% carbamide peroxide (CP) gel with carbopol (positive control) and bleaching with experimental 10% CP gels with natrosol and aristoflex. Participants used a palatal appliance containing bovine enamel/dentin specimens for 15 days. From day 2 to day 15, specimens were bleached extraorally. The bleaching gel was applied according to the groups for four hours. When the bleaching gel was removed, the palatal appliance was inserted again in the participants’ mouth until the next day for another bleaching application. This procedure was repeated for 14 days and on day 15, surface microhardness (SMH), color (ΔE*_ab_ and ΔE_00_), surface roughness (Ra), scanning electron microscopy (SEM), and energy-dispersive X-ray spectrometry (EDS) analyses were performed and data were subjected to statistical analysis.

**Results:**

Neither salivary flow nor thickeners influenced ΔE^*ab^ and ΔE_00_ results. Carbopol had the lowest SMH, the highest Ra, and the lowest Ca% among all groups. For normal flow, natrosol and aristoflex had higher SMH. For low flow, aristoflex had higher SMH and natrosol and aristoflex had lower Ra. Aristoflex had higher Ca% and Ca/P and differed from carbopol for normal flow.

**Conclusion:**

For normal flow, 10% CP gels with natrosol and aristoflex caused fewer surface changes, and for low flow, only the 10% CP gel with aristoflex.

## Introduction

Tooth bleaching induces important structural changes in human tooth enamel, such as changes in tooth surface morphology, the distribution of hydroxyapatite crystals, increased porosity, decreased surface and subsurface microhardness, calcium loss, and changes in the calcium/phosphate ratio.^1–3^ These changes are possibly related to factors such as pH, the oxidative effect, and the concentration of bleaching agents, or to the composition of bleaching gels, such as thickeners.^4,5^

The thickener used with the carbamide peroxide (CP) gel is carbopol (polymer carboxypolymethylene); however, this thickener has ionic characteristics and acid pH, which contributes to the degradation of the enamel surface; thus, other thickeners have been evaluated. An alternative would be using natrosol (hydroxyethyl cellulose), a polymer with non-ionic characteristics that can be used with acid substances, as it has a stable pH.^6^ Another option would be using aristoflex (ammonium acryloyldimethyltaurate copolymer), as it has a stable acid pH and forms an anionic gel capable of acting as an inert viscous agent in the formulation.^5^

While tooth bleaching results in deleterious effects on enamel, human saliva is able to prevent and reverse changes in dental tissue related to tooth bleaching.^7^ Saliva is responsible for the formation of the acquired pellicle that acts as a diffusing barrier or selectively permeable membrane that prevents direct contact between the bleaching gel and tooth surface. Moreover, when pH is within the physiological limits, saliva is supersaturated with calcium and phosphate, which are minerals that favor the remineralization process.^8^

Due to the essential action of saliva during and after bleaching, it is important to evaluate salivary changes that may interfere in the remineralization capacity of enamel bleached by saliva. Hyposalivation affects approximately 30% of patients aged 20 to 69 years^9^ and studies showed that changes in salivary flow are followed by changes in salivary characteristics, such as pH, protein and electrolyte concentrations, viscosity, and immunoglobulin levels.^10^ Thus, in hyposalivation conditions, there is no way to guarantee the presence of all salivary protection and remineralization that would be expected in patients with normal salivary flow.

This *in situ* study aimed to evaluate the effect of the application of experimental bleaching gels on the dental enamel of participants with normal and low salivary flow. The null hypotheses tested were: (1) salivary flow would not influence substrate changes caused by different bleaching gels with different thickeners; and (2) thickeners would not influence chemical and physical substrate characteristics.

## Methodology

### Experimental design

**Figure 1:**
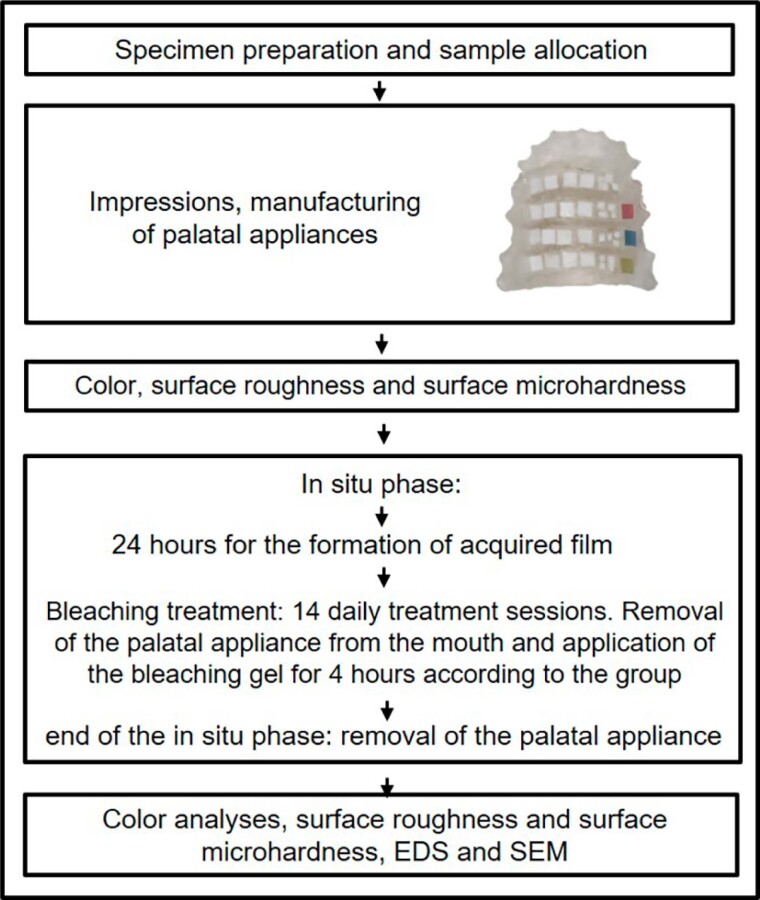
Study diagram

This *in situ* study tested salivary (flow two levels: normal salivary flow and low salivary flow), and bleaching gels [four levels: no bleaching (negative control), commercial CP gel with carbopol (positive control), experimental CP gel with natrosol, and experimental CP gel with aristoflex]. The analyses performed were surface microhardness (SMH) (before and after bleaching), surface roughness (Ra) (before and after bleaching), color analysis by reflectance spectrophotometry (ΔE^*^_ab_ and ΔE_00_), energy-dispersive X-ray spectrometry (EDS), and scanning electron microscopy (SEM). Figure 1 shows the study diagram.

### Participants and ethical aspect

This study was conducted according to the Declaration of Helsinki and approved by the local Research Ethics Committee (process no. 96044418.8.0000.5418). The number of participants was estimated using the G*Power 3.1.7 program, considering a minimum power of 0.80 for the main effects, interaction with a 5% significance level, and average effect size according to previous studies.^11^ The minimum number of participants with low and normal salivary flow was 14 each, totaling 28 participants. Seven men and seven women were selected for each type of salivary flow. The informed consent form was signed by all participants. Participants with low salivary flow (45–65 years old) underwent head and neck radiotherapy during cancer treatment at least two years before the study. The inclusion criterion for those participants was reduced salivary flow rate (stimulated flow rate <0.8 ml/min).^12^ For participants with normal flow rate (25–35 years old), the inclusion criterion was stimulated flow rate >1.0 ml/min.^12^ All participants had to meet all other inclusion criteria: not having active caries and periodontal disease, not using orthodontic appliance, having at least 50% of their upper dental arch (eight teeth) for fixing the palatal device, being non-smokers, and not being pregnant or breastfeeding. No participant took any medication that affected salivary flow rate, such as antidepressants, anxiolytics, antihypertensives, or antiallergics.

### Analysis of salivary parameters

Participants were instructed to chew a piece (1 g) of parafilm (Parafilm M, Pechiney Plastic Packaging Inc., Chicago, IL, USA). The saliva produced in the first 30 seconds was discarded and then, during the next five minutes, participants dispensed the saliva produced in a meter test tube.^13^ Collections were carried out from 9 am to 11 am or from 2 pm to 4 pm and should also take place at least one hour after eating and after oral hygiene to minimize the effects of daily variability in the salivary composition.^14^ Immediately after collection, salivary flow was estimated by dividing the volume of saliva (considering that 1 ml corresponds to 1 g) by the collection time. Salivary pH was estimated using a pH meter (Orion 290A+, São Paulo, Brazil). To estimate the buffer capacity, 0.5 ml of stimulated saliva was added to 1.5 ml of 0.005 M HCl in a plastic tube that was at rest for five minutes to release CO_2_ from the reaction of the bicarbonate buffer of saliva with the acid. The pH of this mixture was estimated in a previously calibrated parameter as an estimate of the salivary buffer capacity.^15^

### Preparation of specimens

For this study, 4×4×2-mm enamel and dentin specimens were obtained from bovine teeth using a metallographic cutter (IsoMet 1000, Buehler Ltd., Lake Bluff, IL, USA). For the surface microhardness analysis, 2×2×2-mm enamel and dentin specimens were used, since they are destructive and would prevent the performance of the other analyses proposed in this study. For grinding, regularization, and polishing, decanting silicon carbide sanding discs were used (#1200/2500/p4000, Buehler Ltd., Lake Bluff, IL, USA), and the tested surfaces were polished with felts (TCT and TWI; Arotec, Cotia, SP, Brazil) associated with diamond pastes with decreasing granulation (1 and ¼ μm; Arotec, Cotia, SP, Brazil). After polishing, specimens were sterilized with ethylene oxide and stored in water at 4°C until use.

### Sample allocation

In order to reduce intra-voluntary variability, in the palatal appliance, each participant received four 4×4-mm and 2-mm-thickness specimens (1 mm enamel and 1 mm dentin) from each group for non-destructive analyses (color analysis, surface roughness, SEM, and EDS) and four 2×2-mm and 2-mm-thickness specimens (1 mm enamel and 1 mm dentin) for the surface microhardness analysis. The randomization of specimens between groups was performed by the microhardness analysis (for 2×2×2-mm specimens) and color analysis (for 4×4×2-mm specimens), so that there was no statistical difference between the initial values, in order to reduce the initial variability between groups. After analyses, the average of the values obtained was estimated for each analysis and at the end, a value per analysis and per group was considered for each participant (n=14).

### Compounding of experimental bleaching gels

**Figure 2:**
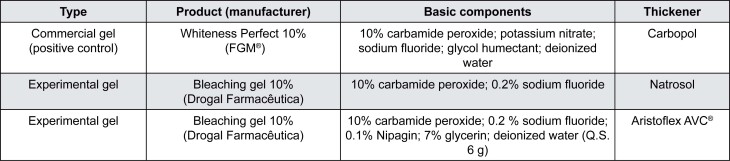
Bleaching gels used in the study, according to the manufacturer’s information

A commercial gel with carbopol (Whiteness Perfect 10%, FGM, Joinville, Brazil) was used as positive control. Bleaching gels with natrosol or aristoflex were compounded (Drogal Farmacêutica LTDA, Piracicaba, SP, Brazil). Figure 2 shows the composition of each bleaching gel.

### *In situ* phase

Plaster models were molded with alginate from the participants’ upper dental arch and palatal devices were made of acrylic resin. A fluoride-free toothpaste was used during the experimental period to avoid the use of any substance that could compromise the remineralization capacity of saliva. The placebo toothpaste was compounded (Drogal Farmacêutica LTDA, Piracicaba, SP, Brazil) using glycerin, silica, carboximethyl cellulose, water, methyl p, saccharin, titanium dioxide, sodium lauryl sulfate, and mint oil. The use of other oral hygiene products was not allowed during the experimental period. The total study period was 15 days. In the four days before the experimental phase, participants also used a non-fluoridated toothpaste to avoid the presence of residual fluoride in saliva.

Participants used intraoral appliances for one day before bleaching sessions for the formation of the acquired pellicle. There were 14 daily treatment sessions. The number of applications was determined according to the literature, mainly from studies that evaluated bleaching gels with different thickeners.^5,6,16^ Participants were instructed to remove the palatal appliance and apply the bleaching gel according to their group. The negative control group did not receive a bleaching gel. A thin layer of bleaching gel was applied and removed after four hours with flexible cotton tipped rods. Then, samples were washed with water and the palatal appliance was reinserted into the participants’ mouth.

### Color analysis

Analyses were performed in a light chamber (GTI MiniMatcher MM1e, GTI Graphic Technology Inc., Newburgh, NY, USA) in order to standardize ambient light. The equipment used was a spectrophotometer (CM 700D, Konica Minolta, Osaka, Japan). The values obtained were quantified in three coordinates of the CIE Lab System (L*, a*, b*). The color analysis was performed at the initial time and 24 hours after the in situ experimental period. The color change was calculated using the following equation: $\Delta {\text{E}}^{*}{}_{\text{ab}}={\left[{\left(\text{L}1-\text{L}0\right)}^{2}+{\left(\text{a}1-\text{a}0\right)}^{2}+{\left(\text{b}1-\text{b}0\right)}^{2}\right]}^{½}$ and by the ΔE_00_ formula:^17^


$$\Delta E_{\infty }={\left[{\left(\frac{\Delta L^{\prime }}{K_{L}S_{L}}\right)}^{2}+{\left(\frac{\Delta C^{\prime }}{K_{C}S_{C}}\right)}^{2}+{\left(\frac{\Delta H^{\prime }}{K_{H}S_{H}}\right)}^{2}+R_{T}\left(\frac{\Delta C^{\prime }}{K_{C}S_{C}}\right)\left(\frac{\Delta H^{\prime }}{K_{H}S_{H}}\right)\right]}^{1/2}$$

### Surface microhardness

Microhardness values were obtained by the arithmetic mean of five indentations carried out in the central region of the specimen with 100 µm distance using a microhardness tester and a Knoop penetrator (HMV-2000, Shimadzu, Tokyo, Japan) with a load of 50 g for five seconds. For each participant, a microhardness value was obtained for each specimen and the mean of these values was estimated to obtain a value per participant and per group. The surface microhardness analysis was performed at the initial time and 24 hours after the *in situ* experimental period.

### Surface roughness

Surface roughness (Ra) was analyzed using a profilometer tester (Surfcorder 1700, Kosaka, Tokyo, Japan) at the initial time and 24 hours after the in situ period. Three different equidistant 1.25-mm scans were measured on the surface of each sample, with a 0.25-mm cut-off and a speed of 0.1 mm/s.

### Energy-dispersive X-ray spectrometry (EDS)

Five samples per group were randomly selected for EDS.^1^ Samples were subjected to vacuum sputtering with carbon (Delton Vaccum, Desk II, Moorestown, NJ, USA). Then, the inorganic content present in substrates was evaluated by a scanning electron microscope (JSM 5600LV, JEOL Ltd., Tokyo, Japan) with typical energies of the order 15kV, at 100× magnification, with PHA deadtime varying from 20% to 25%. The analysis was performed in dental enamel, in five regions per randomly selected specimen to determine Ca and P levels (% by weight) and the ratio between Ca and P. For each sample, results were presented by the average percentage of the chemical elements found on the surface of blocks.

### Scanning electron microscopy (SEM)

Four specimens for each group were randomly selected after all analyses and dehydrated in increasing degrees of ethanol at concentrations of 50%, 60%, 70%, 80%, 90%, and 100% for 20 minutes. Then, they were sputtered with gold (90 seconds; Baltec, MED/JEOL Ltd., Tokyo, Japan) and images of representative areas of specimens at 4000x magnification were obtained using a scanning electron microscope (JSM 5600LV, JEOL Ltd., Tokyo, Japan).

### Statistical analysis

All analyses were performed in the SAS 9.4 software (SAS Institute Inc., Cary, NC, USA, 2012) with a 5% significance level. Descriptive and exploratory data analyses were carried out. Surface microhardness and surface roughness were analyzed by mixed models for repeated measures in the time variable using the Tukey-Kramer test. For surface roughness, the inverse transformation was applied, according to the exploratory analysis. ΔE*_ab_, ΔE_00_, Ca, P, and Ca/P data did not fit in a known distribution and were analyzed by Mann-Whitney nonparametric tests in order to compare participants with normal and low salivary flow. Friedman and Nemenyi tests were performed to compare bleaching gels. Salivary flow was analyzed by the Mann-Whitney test and the buffer capacity and pH were evaluated by the Student t-test.

## Results

### Salivary flow rate, pH, and buffer capacity

Table 1 presents the salivary flow rate, pH, and buffer capacity of each participant. There was a significant difference between groups regarding salivary flow rate (p<0.0001), but not for pH (p=0.8268) and buffer capacity (p=0.6930).

**Table 1:** Mean and standard deviation of salivary flow rate, pH, and buffer capacity of the stimulated saliva for normal and low flows

	Flow rate (ml/min)	pH	Buffer capacity
Normal flow	1.96 (0.29)*	7.50 (0.22)	4.80 (0.78)
Low flow	0.62 (0.12)*	7.52 (0.18)	4.67 (0.80)

* p<0.0001: statistically significant differences between groups (t-test for independent data).

### Color analyses

∆E^*^_ab_ and ∆E_00_ results (Table 2) showed that the experimental gels used differed statistically from the positive control group, but not from the negative control group.

**Table 2:** Mean and standard deviation of surface color analyses (ΔE*_ab_ and ΔE_00_) as a function of salivary flow and bleaching gel

Bleaching gel	ΔE*_ab_		ΔE_00_	
	Salivary flow		Salivary flow	
	Normal flow	Low flow	Normal flow	Low flow
Unbleached	1.58 (0.75)^Ab^	1.56 (0.65)^Ab^	1.13 (0.50)^Ab^	1.17 (0.38)^Ab^
Carbopol gel	5.72 (1.48)^Aa^	5.36 (1.41)^Aa^	4.13 (1.05)^Aa^	3.82 (1.04)^Aa^
Natrosol gel	6.18 (1.39)^Aa^	5.91 (1.40)^Aa^	4.45 (0.93)^Aa^	4.13 (0.99)^Aa^
Aristoflex gel	6.07 (1.28)^Aa^	5.98 (1.12)^Aa^	4.34 (0.89)^Aa^	4.24 (0.80)^Aa^

Different letters (lowercase in the vertical) show significant differences (p≤0.05). The same letter (uppercase in the horizontal) shows no significant differences (p≥0.05).

### Surface microhardness

Table 3 presents the surface microhardness analysis results. All bleached groups showed a decrease in surface microhardness after bleaching and were statistically different from the negative control group (p≤0.05), regardless of the bleaching gel used and salivary flow.

**Table 3:** Means and standard deviation of surface microhardness (SMH) as a function of salivary flow and bleaching gel

Bleaching gel	Salivary flow			
	Normal flow		Low flow	
	Before	After	Before	After
Unbleached	325.33 (9.68)^Aa^	323.79 (5.33)^Aa^	328.27 (12.82)^Aa^	327.16 (6.71)^Aa^
Carbopol gel	327.46 (10.36)^Aa^	271.33 (5.17)^Bc^	325.97 (11.94)^Aa^	264.45 (6.07)^Bd^
Natrosol gel	324.60 (7.99)^Aa^	304.19 (4.31)^Bb^	324.36 (12.02)^Aa^	*281.81 (14.06)^Bc^
Aristoflex gel	325.74 (11.30)^Aa^	305.64 (2.32)^Bb^	324.85 (11.99)^Aa^	306.08 (5.16)^Bb^

* This value differs from the group with normal salivary flow, under the same bleaching gel and time conditions (p≤0.05). Different letters (uppercase in the horizontal, comparing between the two times for the same salivary flow, and lowercase in the vertical) show significant differences (p≤0.05). p(salivary flow)=0.0729; p(bleaching gel)<0.0001; p(salivary flow × bleaching gel)=0.0007; p(time)<0.0001; p(salivary flow × time)=0.0168; p(bleaching gel × time)<0.0001; p(salivary flow × bleaching gel × time)=0.0077.

For normal flow, after bleaching, the negative control group had the lowest values and was statistically different from groups that used experimental gels (p≤0.05). Groups that used natrosol and aristoflex gels showed the highest values and did not differ statistically between themselves.

For low flow, after bleaching, the negative control group had the lowest values and was statistically different from all other groups (p≤0.05). The natrosol group had intermediate values and was statistically different from the other bleached groups (p≤0.05). Among the bleached groups, the highest values after bleaching were found in the aristoflex group, which was statistically different from the other bleached groups (p≤0.05).

Comparing salivary flows, for low flow, the natrosol group had lower values after bleaching when compared with the same bleaching gel during the same time for normal flow (p≤0.05). In other groups, no statistically significant differences were found between salivary flows under the same bleaching gel and time conditions (p≥0.05).

### Surface roughness

Surface roughness results (Table 4) show that all bleached groups presented increased roughness values after bleaching and were statistically different from the negative control group (p≤0.05).

**Table 4:** Means and standard deviation of surface roughness (Ra) as a function of salivary flow and bleaching gel

Bleaching gel	Salivary flow			
	Normal flow		Low flow	
	Before	After	Before	After
Unbleached	0.084 (0.004)^Aa^	0.083 (0.004)^Ab^	0.081 (0.008)^Aa^	0.080 (0.009)^Ac^
Carbopol gel	0.080 (0.004)^Ba^	0.124 (0.008)^Aa^	0.083 (0.006)^Ba^	*0.165 (0.005)^Aa^
Natrosol gel	0.081 (0.005)B^Ba^	0.116 (0.004)^Aa^	0.080 (0.006)^Ba^	0.121 (0.011)^Ab^
Aristoflex gel	0.082 (0.006)^Ba^	0.109 (0.004)^Aa^	0.081 (0.006)^Ba^	0.107 (0.006)^Ab^

* This value differs from the group with normal salivary flow, under the same bleaching gel and time conditions (p≤0.05). Different letters (uppercase in the horizontal, comparing the two times for the same salivary flow, and lowercase in the vertical) show significant differences (p≤0.05). p(salivary flow)=0.2545; p(bleaching gel)<0.0001; p(salivary flow × bleaching gel)=0.0003; p(time)<0.0001; p(salivary flow × time)=0.0196; p(bleaching gel × time)<0.0001; p(salivary flow × bleaching gel × time)=0.0190.

For normal flow, after bleaching, no statistically significant differences were found between the bleaching gels used (p≥0.05). For low flow, after bleaching, the positive control group showed the highest values and was statistically different from all other groups (p≤0.05). The natrosol and aristoflex groups had the lowest values and did not differ statistically between themselves.

Comparing salivary flows, for low flow, the positive control group had higher values after bleaching when compared with the same bleaching gel at the same time for normal flow (p≤0.05). In other groups, no statistically significant differences were found between salivary flows under the same of bleaching gel and time conditions (p≥0.05).

### Elemental levels (wt%) and EDS

Table 5 presents Ca% values. For normal flow, statistically significant differences were found between groups (p=0.0070). The positive control group had the lowest Ca% values and was statistically different from other bleached groups; however, it was not statistically different from the negative control group.

**Table 5:** Mean and standard deviation of elemental levels (wt%) of the enamel surface according to the group, as a function of salivary flow and bleaching gel

Bleaching gel	Ca%		P		Ca/P	
	Salivary flow		Salivary flow		Salivary flow	
	Normal flow	Low flow	Normal flow	Low flow	Normal flow	Low flow
Unbleached	71.11 (0.14)^Aab^	71.06 (0.23)^Aa^	28.28 (0.12)^Aa^	28.17 (0.24)^Aa^	2.51 (0.02)^Ab^	2.52 (0.03)^Aa^
Carbopol gel	70.85 (0.18)^Ab^	70.95 (0.25)^Aa^	28.15 (0.15)^Aab^	28.19 (0.32)^Aa^	2.52 (0.02)^Aa^	2.52 (0.03)^Aa^
Natrosol gel	71.34 (0.17)^Aa^	71.15 (0.19)^Aa^	28.06 (0.13)^Aab^	28.14 (0.24)^Aa^	2.54 (0.02)^Aab^	2.53 (0.03)^Aa^
Aristoflex gel	71.65 (0.64)^Aa^	71.21 (0.25)^Aa^	27.60 (0.68)^Bb^	28.10 (0.14)^Aa^	2.59 (0.09)^Aa^	2.54 (0.02)^Aa^

Different letters (uppercase in the horizontal and lowercase in the vertical) show significant differences (p≤0.05).

P% values (Table 5) show that significant differences were found between salivary flows in the aristoflex group, which had higher values for low flow (p=0.0472). For normal flow, statistically significant differences were found between groups (p=0.0211). The aristoflex group had the lowest P% values and was statistically different from the negative control group.

For Ca/P% (Table 5), for normal flow, statistically significant differences were found between the bleached groups (p=0.0184). The positive control group did not differ statistically from the negative control group. The aristoflex group showed the highest values and was different statistically from the negative and positive control groups. For low flow, no statistically significant differences were found between all bleached groups (p=0.3647).

### Scanning electron microscopy (SEM)

**Figure 3:**
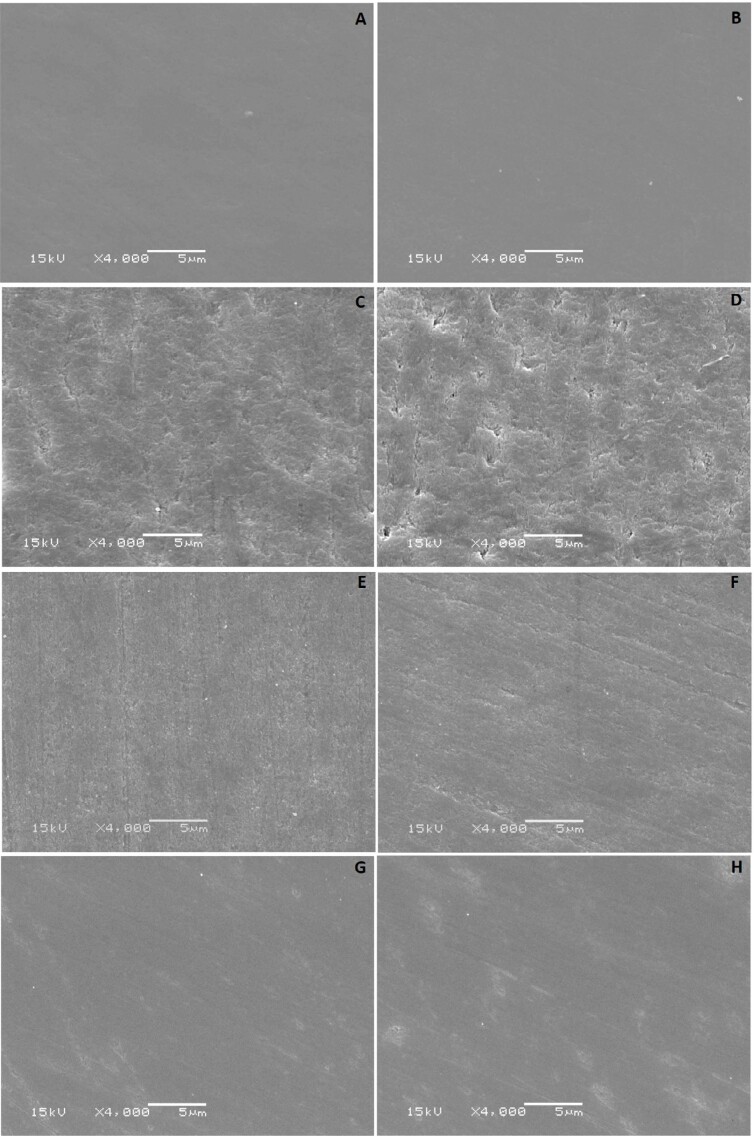
Representative SEM images (4000x) of specimens according to the group: (A) unbleched in normal flow, (B) unbleached in low flow, (C) carbopol in normal flow, (D) carbopol in low flow, (E) natrosol in normal flow, (F) natrosol in low flow, (G) aristoflex in normal flow, (H) aristoflex in low flow

Figure 3 shows SEM images. The positive control group had higher roughness when compared with other bleached groups. For low flow, this group (Figure 3D) showed greater depth and extent of demineralization than the same bleaching gel for normal flow (Figure 3C). The natrosol group showed intermediate surface changes when compared with other bleached groups. For low flow, this group showed higher roughness and lower depth (Figure 3F). For normal flow, a greater depth of demineralization was observed, however, this demineralization was observed in specific parts of the enamel (Figure 3E). The aristoflex group showed fewer surface changes and less evidence of dissolution among all bleached groups (Figure 3G and 1H). Moreover, aristoflex was the only bleaching gel with the same surface characteristics for normal and low flows.

## Discussion

The first null hypothesis was rejected because, according to the bleaching gel used, salivary flow influenced the results of the analyses performed in this study, except for color analysis. The second null hypothesis was rejected because for low salivary flow, experimental bleaching gels with different thickeners caused less deleterious effects on the physical and chemical properties of enamel; however, for normal salivary flow, no differences in surface roughness were found between thickeners.

Salivary flows and the different thickeners used did not interfere in the bleaching efficacy, as for all color analysis performed, the bleached groups did not differ statistically between them, but differed from the negative control group (Table 2). These results are due to the presence of CP in the formulation of all bleaching gels used. Moreover, they all used the same bleaching agent at the same concentration, thus, the only difference between them was the thickener. Carbamide peroxide releases hydrogen peroxide, which dissociates into free radicals, such as oxidizing long-chain organic chromophore molecules that are responsible for the dental tissue color, promoting tooth bleaching.^18^

In this study, for both salivary flows, bleaching with 10% CP led to a significant decrease in enamel microhardness (Table 3) and increase in enamel roughness (Table 4), regardless of the thickener used, when compared with the negative control group. Changes in physical properties were probably associated with demineralization effects, which are caused by the diffusion of the hydrogen peroxide released by the interaction between the dissociation of CP and acid pH from the bleaching gel.^3^ Changes inevitably result from hydrogen peroxide, as oxygen radicals interact with the tooth surface in a non-specific way, regardless of the thickener used.^5^ Moreover, when the initial and final values were compared, saliva was not able to recover enamel microhardness and roughness, regardless of salivary flow. This result may be associated with the fact that thickeners may interfere in the interaction between tooth surface and saliva. A study^4^ showed that thickeners were able to interact with the tooth structure due to their bioadhesive capacity. This capacity is related to possible ionic bonds between polymers: negatively charged centers form a “pellicle” of polymer deposited on the tooth structure, that is, a polymer layer capable of working as a barrier and preventing salivary remineralization,^5^ which justifies some results of this study.

For normal and low salivary flows, the carbopol gel (positive control) led to lower enamel microhardness values when compared with other thickeners (Table 3). This thickener has acid pH and can contribute to demineralization during tooth bleaching.^5^ More acid compounds may be responsible for a higher demineralization.^19^ For low flow, the carbopol gel led to the highest enamel roughness values and differed statistically from other thickeners. Moreover, differences in surface roughness were found between the two salivary flows. For low flow, the carbopol gel led to the highest enamel roughness after bleaching and differed statistically from the values found for normal flow (Table 4). The remineralization process modulated by saliva occurs irregularly, inducing the reorganization of enamel prisms, which can increase roughness.^19^ Moreover, the bioadhesive capacity of carbopol and its interaction with the enamel surface result in the formation of a very strong and thick layer of polymers, showing the great affinity of this thickener to the tooth structure.^4^ Due to its strong calcium-binding capacity, carbopol inhibits the incorporation of hydroxyapatite crystals during remineralization.^20^ This could result in the need for a larger volume of saliva to promote enamel remineralization, which justifies the worst results for low salivary flow.

Moreover, the differences in the effect of carbopol found between salivary flows may be associated with the fact that changes in salivary flow are followed by changes in salivary characteristics, such electrolyte concentrations.^10,21^ The supersaturation of the same electrolyte, such as fluoride, calcium, and phosphate, in saliva is critical to the remineralization process.^22^ Changes in salivary flow can result in changes in the amount of protein in saliva. Proteins, such as statherins, histatins, cystatins, and proline-rich proteins, bind to hydroxyapatite to help control the crystalline growth of enamel by limiting mineral loss during demineralization and allowing the penetration of minerals into the enamel for remineralization.^23^ Thus, changes in salivary characteristics in individuals with low salivary flow may result in a lower remineralization capacity.

EDS analysis supported microhardness and roughness results for the positive control gel. Regarding Ca%, for normal flow, although no group differed statistically from the negative control group, the positive control group showed the lowest values and differed statistically from the other groups. Moreover, this group had the lowest Ca/P values among the thickeners evaluated (Table 5). In tooth bleaching, mineral dissolution occurs with a loss of calcium and phosphorus.^24^ The demineralization process begins with the loss of calcium ions from apatite crystals on the enamel surface, which alters its microhardness.^24^ SEM images confirmed the results found for the positive control group, since they showed a higher level of superficial demineralization compared with other thickeners. Moreover, it was possible to identify that for low salivary flow (Figure 3D), carbopol led to greater depth and extent of demineralization than the same thickener for normal salivary flow (Figure 3C).

For normal salivary flow, the natrosol and aristoflex groups showed the best microhardness results and differed from the positive control group (Table 3). These results were in accordance with previous studies.^5,19^ The natrosol and aristoflex groups had the highest Ca% values and did not differ between them for normal flow (Table 5). Aristoflex and natrosol have stable pH and are capable of forming gels with non-ionic characteristics,^6,25^ which probably resulted in a lower level of demineralization. Moreover, the normal flow condition provided saliva without changes in its characteristics, making it able to act normally and favoring remineralization after bleaching. In participants with normal flow, it was possible to observe higher Ca/P% values for both groups and the aristoflex group differed statistically from the positive and negative control groups (Table 5). Changes in the Ca/P ratio show changes in the inorganic components of hydroxyapatite.^26^ This may have happened by the deposition of salivary calcium on the enamel surface during the remineralization process, which would result in an increase in Ca and a decrease in P, changing the Ca/P% ratio for this group.

For low flow, natrosol did not have the same performance as it had for normal flow, confirming the importance of interaction between salivary flow and this thickener. Although aristoflex and natrosol had the lowest enamel roughness values for low salivary flow (Table 4), natrosol showed intermediate microhardness among the thickeners evaluated (Table 3). Moreover, a difference in microhardness was observed between salivary flows for this thickener. For low flow, the use of natrosol resulted in lower microhardness when compared with its use for normal flow (Table 3). SEM images confirmed this data, since it was possible to observe for low salivary flow a greater extent of demineralization whereas for normal flow, demineralization was localized (Figure 3). Natrosol also interacted with the tooth surface by forming a layer on enamel. This layer is not resistant to low pH^4^ in the same way as hydrogen peroxide pH and allows saliva to come into contact with the enamel surface.^4^ However, natrosol is capable of forming complexes with calcium and/or phosphate ions,^27^ which may have left these elements unavailable for remineralization. Moreover, changes in salivary flow are followed by changes in the calcium and phosphate concentration in saliva, resulting in a lower remineralization capacity. The association of these two factors could explain the results found for this thickener for low salivary flow.

For low salivary flow, the aristoflex group showed the highest microhardness values (Table 3). Moreover, this group did not differ in roughness values from the natrosol group for low flow, but had the lowest roughness among all groups (Table 4). Besides the characteristics of this gel, which may have resulted in minor surface changes after bleaching, these results may be related to the good interaction between this gel and the tooth surface. Unlike the other thickeners evaluated in this study, aristoflex form bonds that are not strong enough or resistant to acid,^4^ allowing the tooth surface to be free for and available to salivary remineralization.^5^ This fact justifies the best performance of this thickener regarding surface microhardness for low flow compared with the other thickeners evaluated. Moreover, no differences were found between salivary flows. This result was confirmed by SEM images, as the aristoflex group was the only one that did not show differences between salivary flows (Figure 3). The combination between aristoflex and fluoride tended to reduce the loss of surface hardness.^4^ As a positively charged molecule, aristoflex is able to attract fluoride ions existing in saliva to interact with its positive parts, helping in the completely beneficial interaction between fluoride and the enamel surface.^4^ Due to this property, the presence of saliva and its fluoride ions, regardless of the volume, was sufficient to promote the remineralization effects of enamel.

## Conclusion

The thickeners evaluated interfered in the remineralization capacity of saliva after bleaching. The carbopol bleaching gel promoted the greatest changes in the mineral content of enamel for the two salivary flows. For normal salivary flow, aristoflex or natrosol gels showed the best results for enamel microhardness. For normal and low salivary flows, bleaching with a aristoflex gel showed minor surface changes.
